# Biomass-derived functional porous carbons as novel electrode material for the practical detection of biomolecules in human serum and snail hemolymph

**DOI:** 10.1038/srep10141

**Published:** 2015-05-22

**Authors:** Vediyappan Veeramani, Rajesh Madhu, Shen-Ming Chen, Bih-Show Lou, Jayabal Palanisamy, Vairathevar Sivasamy Vasantha

**Affiliations:** 1 Electroanalysis and Bioelectrochemistry Lab, Department of Chemical Engineering and Biotechnology, National Taipei University of Technology, No. 1, Section 3, Chung-Hsiao East Road, Taipei 106, Taiwan, ROC; 2 Chemistry Division, Center for General Education, Chang Gung University, Tao-Yuan, Taiwan; 3 Department of Physical Sciences, Bannari Amman institute of Technology, Sathyamangalam-638401, Erode, Tamilnadu, India; 4 Department of Natural Products Chemistry, School of Chemistry, Madurai Kamaraj University, Madurai, Tamil Nadu-625 021, India

## Abstract

The biomass-derived activated carbons (ACs) have been prepared with high surface areas up to 793 m^2^ g^−1^ is by ZnCl_2_ activation at three different temperatures, *viz.* AC700, AC800, and AC900. The AC samples were characterized by a variety of analytical and spectroscopy techniques. The as-synthesized ACs were adopted for the simultaneous electrochemical detection of ascorbic acid (AA), dopamine (DA), and uric acid (UA). For comparison, reduced graphene oxide (RGO) was employed for the proposed sensor. The high surface area, modulated pore size and the presence of oxygen surface functional groups like heteroatoms (83.427% C, 1.085% N, 0.383% S, and 0.861% H) in the biomass-derived AC is found to be responsible for the excellent catalytic activities of biomolecules. Fascinatingly, the facile sensor further used to detect biomolecules levels in the snail hemolymph and human blood serum. Notably, the obtained analytical parameters for the biomolecules detection over the AC modified GCE, outperforming several carbon-based modified electrodes in literatures.

Multidimensional (0, 1, 2 and 3D) carbon based materials have been extensively utilized for various applications, such as adsorbents, supercapacitors, batteries, and electrodes^1^,^2^,^3^,^4^,^5^. Among them, graphene is a top candidate in various electrochemical aspects. However, its preparation from graphite is an intricate procedure which may lead to an explosion during the oxidation of graphite. Likewise, the preparation of carbon nanotubes (CNTs) also has practical difficulties because of their complicated instrumental setup. Besides, the preparation of activated carbons (ACs) from bio-wastes are simple, environmentally friendly and cost-effective. In evidence, ACs prepared from various bio-wastes have been widely used in numerous applications, owing to their unique properties, such as ultra high surface area, micro-mesopore volume, low toxicity, excellent chemical stability, electrical conductivity, and presence of oxygen surface functional groups like heteroatoms^5^,^6^,^7^,^8^,^9^. Notably, the availability of literature reports on ACs for electrochemical sensor application is scarce. Moreover, the AC from pumpkin stems has not been explored much, a very few reports available on ACs prepared by using phosphoric acid activation towards the Phenol and Chlorophenol Adsorption^10^,^11^.

Ascorbic acid (AA) is a vital vitamin in the human diet, and it is used as antioxidant and treatment of cancer, common cold, mental illness and AIDS^12^,^13^. Likewise, Dopamine (DA) is an important neurotransmitter which is widely distributed within the mammalian central nervous system (brain, renal, hormonal and cardiovascular systems, etc). Low levels of DA may lead to many diseases (Parkinson’s, schizophrenia, etc.)^14^,^15^,^16^,^17^. In addition, uric acid (UA) is the end product of primary purine metabolite, and its abnormal concentration level in the human body symptomatic several diseases, such as hyperuricemia, leukemia and pneumonia^18^,^19^. Moreover, it is well known that the AA, DA, and UA coexist in the central nervous system and serum. However, the simultaneous determination with selectivity and sensitivity is impossible by using solid electrodes due to their overlapping oxidation peak potentials^20^, insufficient surface area, and also many research have been focused to separate their peak potentials^21^. In particular, carbon-based nanomaterials have been widely used for the simultaneous electrochemical detection of AA, DA and UA with enhanced sensitivity^4^,^22^,^23^.

Herein, we report a novel and efficient electrodes for the simultaneous and selective determination of AA, DA and UA with enormous catalytic activities by using biomass-derived ACs for the first time. Notably, the obtained analytical parameters of biomolecules detection over the as-synthesized ACs, overwhelming the several carbon-based electrodes.

## Results and Discussion

**Figure 1:**
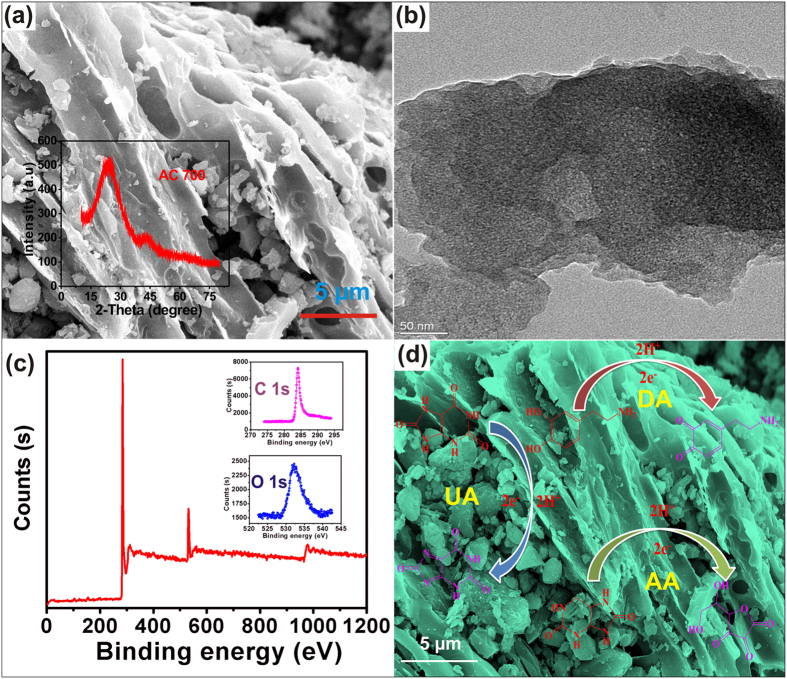
(**a**) FE-SEM image of AC700; inset: XRD pattern of AC700. (**b**) HR-TEM image of AC700. (**c**) XPS spectra of AC700. (**d**) The possible reaction mechanism of the biomolecules.

Figure 1 shows the representative FE-SEM, FE-TEM, XRD, and XPS pattern of the as-synthesized AC. As shown in Fig. 1a, the FE-SEM image of AC700 displays a bundle-like porous morphology, different from the structure of RGO (Figure S1a) and unactivated carbon (Figure S1b). Further FE-TEM (Fig. 1b) study revealed that as-synthesized AC700 contains the mesoscopic pores with short-range ordering, indicating the presence of multi-dimensional wormhole-like pore structure^24^,^25^. The XRD pattern of AC700 (Fig. 1a inset) shows two broad diffraction peaks at 23° and 43°, corresponding to the (002) and (101) plane reflection and reveals the amorphous behavior of the AC^6^. In addition, Fig. 1c shows the XPS survey spectra of AC700 exhibits the peaks corresponding to carbon, and oxygen. Furthermore, CHNS elemental analysis was carried out to confirm the presence of heteroatoms in AC surface, and they are determined to be 83.427% C, 1.085% N, 0.383% S and 0.861% H (see Table S1)^1^,^5^. Owing to the lower concentrations of heteroatoms, the XPS spectra has failed to show their corresponding peaks. However, the main advantage of the AC is the presence of heteroatoms like nitrogen and sulfur in nature without any doping, it may due to the biowaste precursors contain some small amounts of heteroatoms naturally, which may lead to the enhanced electrochemical behavior. Figure S1c and Figure S1d shows the N_2_ sorption studies of the ACs and unactivated carbon, respectively. The unactivated carbon provides a surface area of about 47.76 m^2^ g^−1^ was calculated by the Brunauer–Emmett–Teller (BET) model. Using the BJH model, the calculated pore volume was 0.06 cm^3^ g^−1^ as shown in Figure S1d. Besides, the ZnCl_2_ activated carbons provided the excellent surface areas of AC700, AC800, and AC900 are 793.2, 715.1, and 779.7, m^2^g^−1^ with pore volumes of 0.4, 0.35, and 0.36 cm^3^ g^−1^, respectively^1^,^5^. Furthermore, the pore size distribution curves (Figure S2a) designate the ACs are having well developed micro-mesopores. In order to investigate the structure of the as-prepared carbon materials, the Raman spectroscopy were performed. As shown in Fig. S2b, the activated carbons were showed the two sharp peaks at 1320 (D band), and 1592 (G band), which corresponds to the sp2 carbon and also the intensity of the D band is higher than G band, which designates the AC is amorphous in nature.

**Figure 2:**
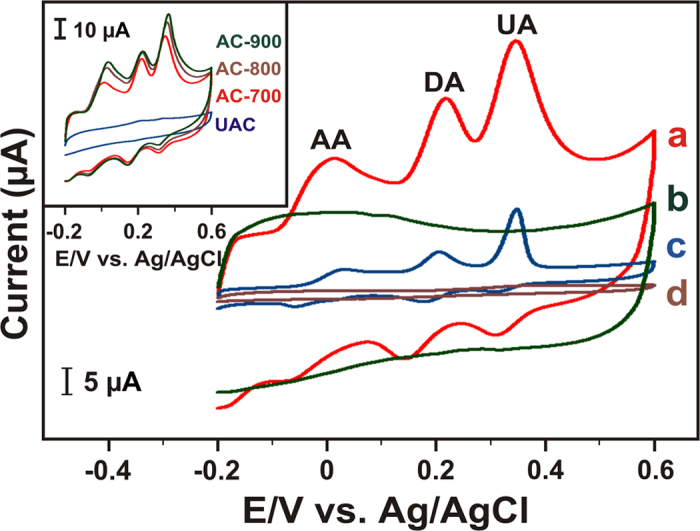
CVs obtained at AC700 (**a**) blank measurement of AC700 (**b**) RGO (**c**) and bare (**d**) modified GCEs in 0.1 M PBS (pH 7), which contained a mixture of 30 μM AA + 10 μM DA +20 μM UA concentrations. Inset: CV curves recorded from different ACs (AC700, AC800 and AC900) and unactivated (UAC) modified electrodes. All CV profiles were recorded at a scan rate of 50 mVs^−1^.

The voltammetric responses of different electrodes towards the simultaneous determination of AA, DA and UA mixture were performed by cyclic voltammetry (CV). As shown in Fig. 2, the bare GCE (curve d) fails to separate the three peaks, resulting in a broad and overlapped peak around 0.4 V containing a mixture of 30 μM AA + 10 μM DA + 20 μM UA concentrations in N_2_ saturated PBS at the scan rate of 50 mV s^−1^. As evidenced, the peak potential separation of three biomolecules is impossible at the bare GCE. Besides, we obtained a well-defined oxidation peaks for AA, DA and UA at AC700 modified electrode (curve a), whereas no peaks observed for the blank measurement (curve b), which confirms the obtained peaks are corresponding to the three biomolecules. In order to optimize the suitable AC modified electrode towards AA, DA and UA, CVs were performed at UAC (unactivated carbon), AC700, AC800 and AC900, as shown in Fig. 2 inset. Among them, AC900 and AC800 displays enhanced peak currents, but possess with higher potentials than the AC700 electrode, which reveals the result with its high surface areas of ACs. However, AC700 exhibits a lower overpotentials with good peak to peak separations (AA-DA = 217, DA-UA = 129 and AA-UA = 346) of each analytes with more sufficient current intensities even at lower concentrations of biomolecules. By taking the advantage of the lower over the potentials of biomolecules, we choose the AC700 as an optimized electrode for further studies, and possible reaction mechanism of the biomolecules as shown in Fig. 1d.

As shown in Fig. 2 (curve c), in comparison with RGO (AA-DA = 169, DA-UA = 143 and AA-UA = 312), our AC700-modified GCE exhibits a good peak to peak separation with several times larger current intensities of the three biomolecules (see Table-S2). Evidently, our AC700-modified GCE outperform all other electrodes due to their high surface area with micro/meso pores. The attributed results validates that the AC700-modified GCE possesses a good electrocatalytic activity towards AA, DA and UA. Figure S3 shows the corresponding cyclic voltammograms of AA, DA and UA the AC700-modified GCE with different scan rates in a pH 7 PBS buffer solution at the same concentrations of three molecules. Both peak potential (Ep) and peak current (Ip) is affected by varying the scan rates, and the CVs clearly displays that the oxidation peak currents (I_pa_) were increased with the increasing scan rates, whereas the peak potentials slightly shifts positively. For all of three biomolecules, the anodic currents were linear with the square root of the scan rate (inset to Figure S3) in the range of 50–500 mV s^−1^. The corresponding linear regression equations are, I_Pa_ (μA) = 2.2157x - 10.592 V^1/2^ (mV/s) AA, I_Pa_ (μA) = 1.6543x - 9.0433 V^1/2^ (mV/s) DA, and I_Pa_ (μA) = 1.6609x - 4.1972 V^1/2^ (mV/s) UA, with linear relative coefficients of 0.985, 0.9913 and 0.9915, respectively.

Hence, the kinetics of overall process were controlled by a diffusion process of AA, DA and UA on the surface of AC700-modified GCE. Moreover, the electrode reaction of DA and UA was quasi-reversible as the redox peak potentials vary with the scan rates. Moreover, while increasing the oxidation peaks of three biomolecules, the capacitances also increased, which can be attributed to the pseudocapacitive contribution from the oxygen surface functional groups and heteroatoms as shown in Table S1. These groups can improve the wettability and maximize the electroactive surface area^23^,^26^.

**Figure 3:**
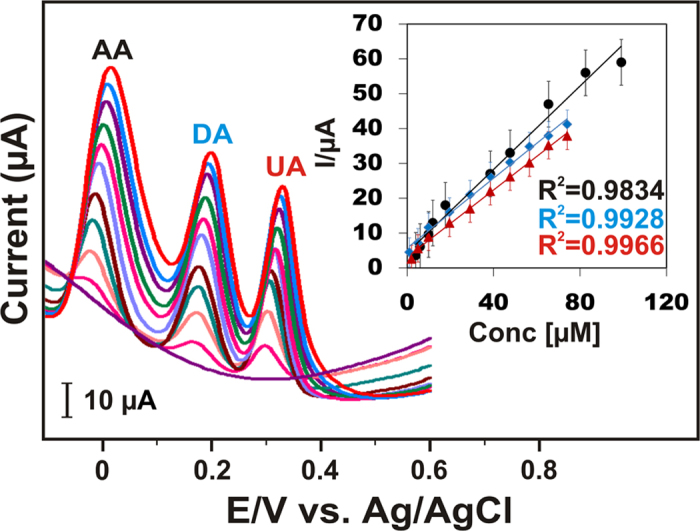
DPV curves of AC700-modified GCE under varied AA, DA, and UA concentrations in 0.1 M PBS (pH 7). Inset; anodic oxidation peak currents *vs* biomolecules concentration.

To investigate the sensitivity for the simultaneous determination of AA, DA and UA, differential pulse voltammetry (DPV) has been adopted which is more sensitive than the other techniques. DPV was carried out in the potential range of −0.2 to 0.6 V at AC700-modified GCE in N_2_ saturated PBS (pH 7.0) solution. Fig. 3 shows the DPVs at different increasing concentrations of AA, DA and UA with well separated anodic peaks. Moreover, peak currents were increased with the increasing concentration of three biomolecules, corresponding linear response curves for three biomolecules are shown in Fig. 3 inset (concentration *vs* increasing current). The calculated sensitivity for AA, DA and UA are, 7.6, 6.3, and 6.1 μA μM^−1^ cm^−2^, and the obtained lower detection limits (LOD) were 2.3, 0.03, and 0.51 μM, respectively. The extraordinary analytical parameters of the reported biosensor at AC700-modified GCE surpassing the previously reported carbon based nanomaterials as shown in Table 1.

**Figure 4:**
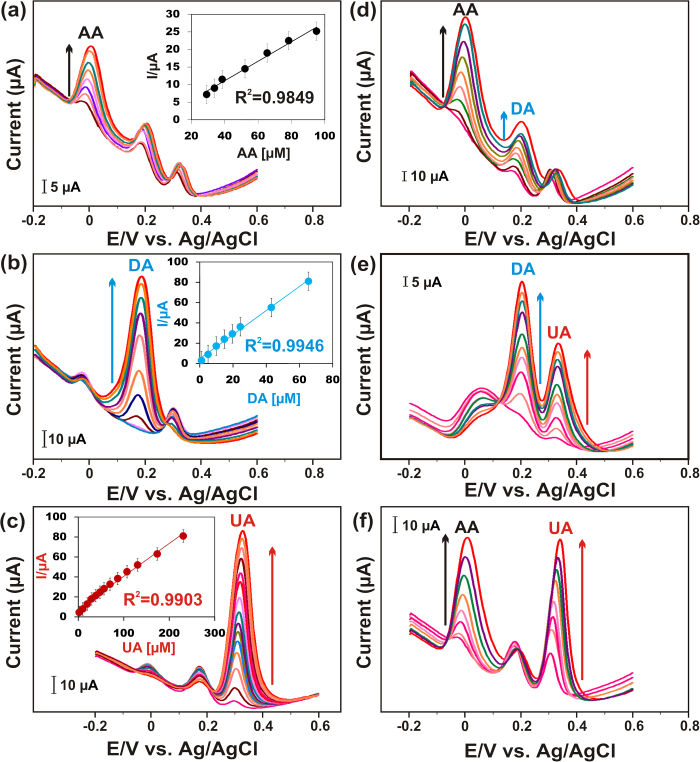
(**a**) DPVs at increasing concentrations of 30–95 μM AA in the presence of 10 μM DA and UA. (**b**) increasing concentrations of 1-65 μM DA in the presence of 33 μM AA and 20 μM UA. (**c**) increasing concentrations of 2-230 μM UA 30 μM AA and 10 μM DA in 0.1 M PBS (pH 7). Insets; anodic oxidation peak currents *vs* biomolecules concentration. (**d-f**) DPVs were performed when the concentrations of two biomolecules changed, whereas the concentration of other biomolecules kept constant.

To investigate the selectivity and cross reactivity of each biomolecule, the DPVs were performed when the concentration of one biomolecule changed, whereas the other two biomolecules kept at constant as shown in Fig. 4. Figure 4a shows the DPVs of AC700-modified GCE at increasing concentrations of 30-95 μM AA in the presence of 10 μM DA and UA, indicating that AA holds good selectivity in the presence of DA and UA. The inset of Fig. 4a shows the corresponding calibration plots of the AA concentrations *vs* peak currents. Similarly, DPV experiments were conducted with DA and UA (Fig. 4b,c), in the presence of 33 μM AA and 20 μM UA or 30 μM AA and 10 μM DA, indicates the I_pa_ of DA and UA is well linear with their increasing concentrations as shown in the insets, respectively. As shown in Table S2, for selective analysis, the calculated detection limits and sensitivities of the three biomolecules are more similar to the simultaneous analyses, which evidenced that our AC modified GCE is more favorable for the simultaneous determination even at their lowest concentration. For the further evidence, as shown in Fig. 4d–f, we have performed the cross reactivity of each analytes, when the concentrations of two biomolecules changed, whereas the concentration of other biomolecule kept constant. Notably, Fig. 4d–f displays the DPVs at increasing concentrations of two biomolecules in the presence of one molecule, indicating that our AC-modified GCE is more feasible towards the selective and simultaneous determination of AA, DA and UA. The obtained results are indirectly indicates the good repeatability, reproducibility of our AC700-modified GCE towards the biomolecules determination in real sample analysis. The attributed results are due to the large surface area, more active sites, and the shapes of isotherms suggest that the high-energy adsorption sites on AC, leading to improved sensitivity for the detection of biomolecules.

In addition, to compare the catalytic oxidation behavior of the as-synthesized AC700 material with RGO, DPVs were performed towards the selective determination of DA in N_2_ saturated PBS (pH 7.0) solution. As shown in Figure S4, for AC modified GCE, the oxidation peak current of DA was observed at 0.162 V and 0.182 V for RGO, and the peak currents linearly increased with increasing concentrations of DA from 5 μM to 230 μM, while 5 μM to 115 μM for RGO. The linear equation of AC700-modified GCE is I_p_/μA = 0.1541 [DA]/μAμM^−1^ + 4.1821, and for RGO is I_p_/μA = 0.068 [DA]/μAμM^−1^ +0.294 and R^2^ = 0.9802. The calculated sensitivity of AC700-modified GCE is 2 μA μM^−1^ cm^−2^ and for RGO-modified GCE is 0.76 μA μM^−1^ cm^−2^. The calculated lower detection limit (LOD) for AC and RGO modified GCEs towards DA detection is 0.045 μM and 0.5 μM, respectively, according to the formula LOD = 3 s_b_/S (where s_b_ is the standard deviation of the blank signal, and S is the sensitivity). The results clearly evidenced that the analytical parameters of the reported AC700-modified GCE in this study are more comparable with RGO-modified GCE (see Table S2). The attributed results are may because of the presence of oxygen surface functional groups and heteroatoms in the as-synthesized AC with high surface area and modulated micro/meso pore sizes. Hence, the AC modified GCE is more suitable for the highly sensitive determination of biomolecules.

**Figure 5:**
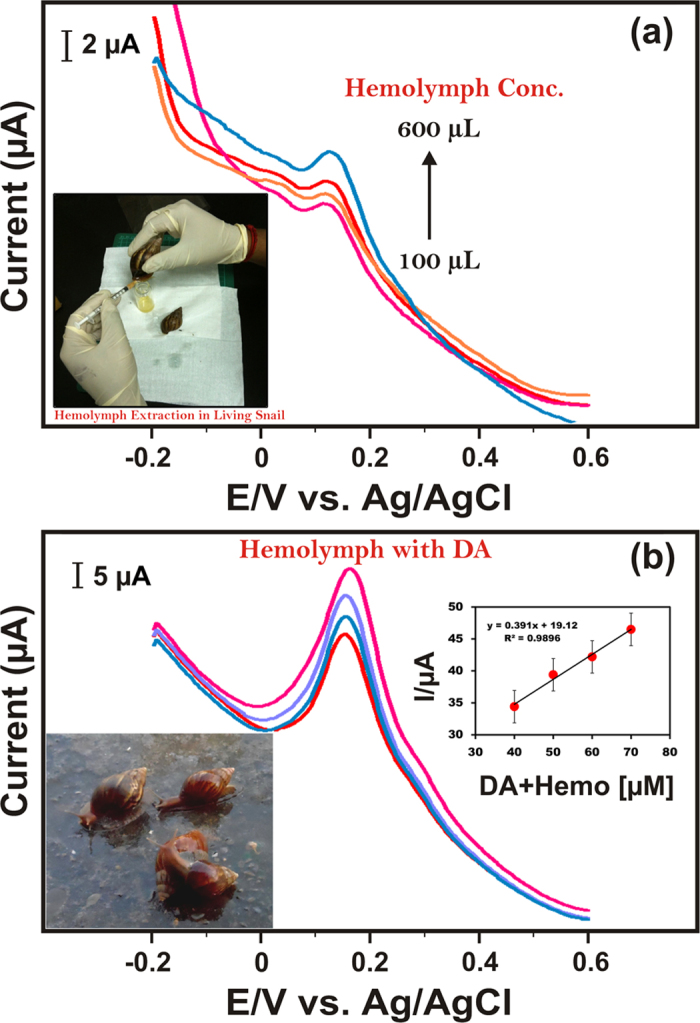
(**a**) DPV curves of AC700-modified GCE under varied snail hemolymph concentrations (100–600 μL), and (**b**) DPV curves of AC700-modified GCE under varied diluted hemolymph with DA concentrations (40 μM-70 μM) in 0.1 M PBS (pH 7). Inset; anodic oxidation peak current *vs* DA with hemolymph concentration (All photographs of Taiwan snails were taken by S.M.C).

In order to prove the real time application of the proposed sensor, snail hemolymph was collected from Taiwan, since it contains dopamine neurons level^27^,^28^,^29^. Interestingly, the 10 ml of snail hemolymph extracts was analyzed by two DPV methods as shown in Fig. 5. First, we directly examined the snail hemolymph extracts without DA concentration. Fig. 5a displays the DPV curves at different concentrations (100–600 μL) of snail hemolymph (as analyte) extracts containing in 0.1 M PBS solution (pH 7.0). Fascinatingly, we obtained the corresponding peak of DA with increasing oxidation peaks while increasing the concentration of snail hemolymph. Furthermore, we have diluted the average quantity of DA concentration in snail hemolymph.

As shown in Fig. 5b (experimental conditions are similar to Fig. 5a), the corresponding oxidation peak currents were increased linearly with increasing DA concentration. Remarkably, we achieved a higher sensitivity (4.9 μA μM^−1^ cm^−2^) and very lower detection limit (0.04 μM), and the obtained results on snail hemolymph extracts are more feasible when compared to the lab sample analysis. In addition, the AC700-modified GCE was further tested for detection of biomolecules in real samples in human serum, the good electrochemical response was observed with the recoveries ranged between 95.2 and 104.4%, as summarized in Table S3. The superior recovery observed for biomolecules in the presence of real samples, indicates the AC-modified electrode is more reliable in practical industrial applications.

In conclusions, the high surface areas of ACs were synthesized by using a simple ZnCl_2_ activation method and characterized by a variety of analytical and spectroscopy techniques is reported. The as-prepared AC sample showed a noteworthy performance for the reported biosensor. The AC700 outperform the other AC modified electrodes and RGO with excellent catalytic activities and quasi-reversible redox behaviors observed during the detection of biomolecules. Notably, the calculated low detection limits and ultra high sensitivity of the biomolecules detection over AC700-modified GCE, overwhelming the numerous carbon-based modified electrodes. In addition, the reported biosensor provides a remarkable performance in snail hemolymph and human blood serum.

## Methods

### Materials

Ascorbic acid, dopamine, and uric acid were obtained from Sigma Aldrich, Zinc chloride (ZnCl_2_) from Wako. All solutions were prepared with deionized water with a resistivity of 18 MΩ/cm Millipore. All chemicals were used as received without any further purification.

### Instrumentation

The cyclic voltammetry (CV) and differential pulse voltammetry (DPV) studies were performed using a CHI900 electrochemical analyzer (CH instruments). A conventional three-electrode cell system was used with an AC-modified glassy carbon electrode (GCE) as the working electrode, an Ag/AgCl (saturated KCl) reference electrode and a platinum wire as the counter electrode. The elemental analysis was carried out using “elementar Vario EL cube” (for CHNS, German). The surface morphology of the film was studied using JEOL field-emission scanning electron microscopy. X-ray diffraction was performed on a Rigaku, MiniFlex II instrument. The N_2_ adsorption-desorption isotherms and pore size distribution was analyzed by using “Micromeritics ASAP 2020”.

### Synthesis of ACs and RGO

The ACs were synthesized by using a simple and eco-friendly method reported elsewhere^30^. Briefly, pumpkin stems (*Cucurbita pepo*) was collected from Dharmapuri (Tamilnadu, India) and washed thoroughly, dried in an oven at 100 °C. The dried pumpkin stems were pulverized and preheated at 150 °C for 2 days. Then, desired amount of preheated sample was activated with 10% of ZnCl_2_ for 24 h under stirring in N_2_ atmosphere at 60 °C, individually. Subsequently, the activated samples were carbonized at various temperatures of 700, 800, and 900 °C for 2 h in N_2_ atmosphere at a heating rate of 10 °C min^−1^ in a tube furnace separately. The carbonized AC powder washed with distilled water and 1 M HCl to remove the Zinc content, and it was referred as pure AC. Finally, the carbonized samples were dried at 100 °C overnight to remove moisture and ground well to get finest powder. For comparison, we have used the electrochemically reduced graphene oxide as reported earlier by us^31^.

### Fabrication of the AC-modified electrode

For the electrochemical biosensor application, the as-synthesized ACs were dispersed in ethanol and sonicated for 2 hours to obtain the stable dispersion. Prior to modification, the GCE surface was carefully polished to a mirror finish with alumina slurry. Then, it was washed with distilled water and ultrasonicated in ethanol-containing water for a few minutes. The *ca.* 6 μl (optimized concentration) of AC dispersion was drop-cast on the pre-cleaned GCE and dried in air oven at 30 °C. Then, the AC modified GCE was gently rinsed a few times with double distilled water to remove the loosely bound AC. The fabricated AC modified electrode was used for further electrochemical experiments, and all the experiments were performed at room temperature in an inert atmosphere.

## Author Contributions

V.V. and R.M. conceived and synthesized the ACs from Pumpkin stem, and they performed the structural, morphological characterizations, and electrochemical experiments for biomolecules detection. R.M. wrote the paper. J.P. gave helps in experiments. The project was finalized by S.M.C., B.S.L. and V.S.V. All authors discussed the results and commented on the manuscript.

## Additional Information

**How to cite this article**: Veeramani, V. *et al*. Biomass-derived functional porous carbons as novel electrode material for the practical detection of biomolecules in human serum and snail hemolymph. *Sci. Rep.* **5**, 10141; doi: 10.1038/srep10141 (2015).

## Supplementary Material

Supplementary Information Supplementary Figures 1-6

## Figures and Tables

**Table 1:** Comparison of analytical parameters of the biomolecules detection over various modified electrodes.

Working electrode	Potential difference (mV)			Linear range (μM)			Detection limit(μM)			Ref.
	**AA-DA**	**DA-UA**	**UA-AA**	**AA**	**DA**	**UA**	**AA**	**DA**	**UA**	
Graphene/carbon fiber electrode	250	130	380	45–1489	0.7–45	0.7–45	24.7	0.5	2	32
Mesoporous carbon nanofiber/graphite electrode	223	149	382	0.1–10	0.05–30	0.5–120	50	0.02	0.2	33
Nitrogen doped porous carbon nanopolyhedra/Glassy carbon electrode	228	124	352	80–2000	0.5–30	4–50	0.7	0.1	0.02	34
Exfoliated flexible graphite paper/ graphite electrode	210	120	330	20–400	0.5–35	0.5–35	2	0.01	0.02	35
Hollow nitrogen doped carbon spheres- reduced graphene oxide/Glassy carbon electrode	252	132	384	50–1200	0.5–90	1–70	0.6	0.01	0.01	36
Electrochemically reduced graphene oxide	240	130	370	500–2000	0.5–60	0.5–60	250	0.5	0.5	37
Single-walled carbon nanohorn/glassy carbon electrode	221	152	383	30–400	0.2–3.8	0.06–10	5	0.06	0.02	38
AC	217	129	346	30-95	1-65	2-230	4.96	0.06	0.75	This work
