# Supplementary material for: Biomass-derived functional porous carbons as novel electrode material for the practical detection of biomolecules in human serum and snail hemolymph
Source: Sci Rep. 2015 May 22;5:10141. doi: 10.1038/srep10141 (PMC4441114; doi:10.1038/srep10141)
Supplement: Supplementary Information — Supplementary Figures 1-6 [file srep10141-s1.pdf]

Supplementary Information

Biomass-derived functional porous carbons as novel electrode material for the practical detection of biomolecules in human serum and snail hemolymph

Vediyappan Veeramani,[†1] Rajesh Madhu,[†1] Shen-Ming Chen,*[1] Bih-Show Lou,*[2] Jayabal Palanisamy,[3] and Vairathevar Sivasamy Vasantha[4]

[1]Electroanalysis and Bioelectrochemistry Lab, Department of Chemical Engineering and Biotechnology, National Taipei University of Technology, No. 1, Section 3, Chung-Hsiao East Road, Taipei 106, Taiwan, ROC.

[2]Chemistry Division, Center for General Education, Chang Gung University, Tao-Yuan, Taiwan.

[3]Department of Physical Sciences, Bannari Amman institute of Technology, Sathyamangalam-638401, Erode, Tamilnadu, India.

[4]Department of Natural Products Chemistry, School of Chemistry, Madurai Kamaraj University, Madurai, Tamil Nadu-625 021, India.

Correspondence and requests for materials should be addressed to S.M.C and B.S.L. (smchen78@ms15.hinet.net&blou@mail.cgu.edu.tw)

[†]These authors contributed equally

Figure S1

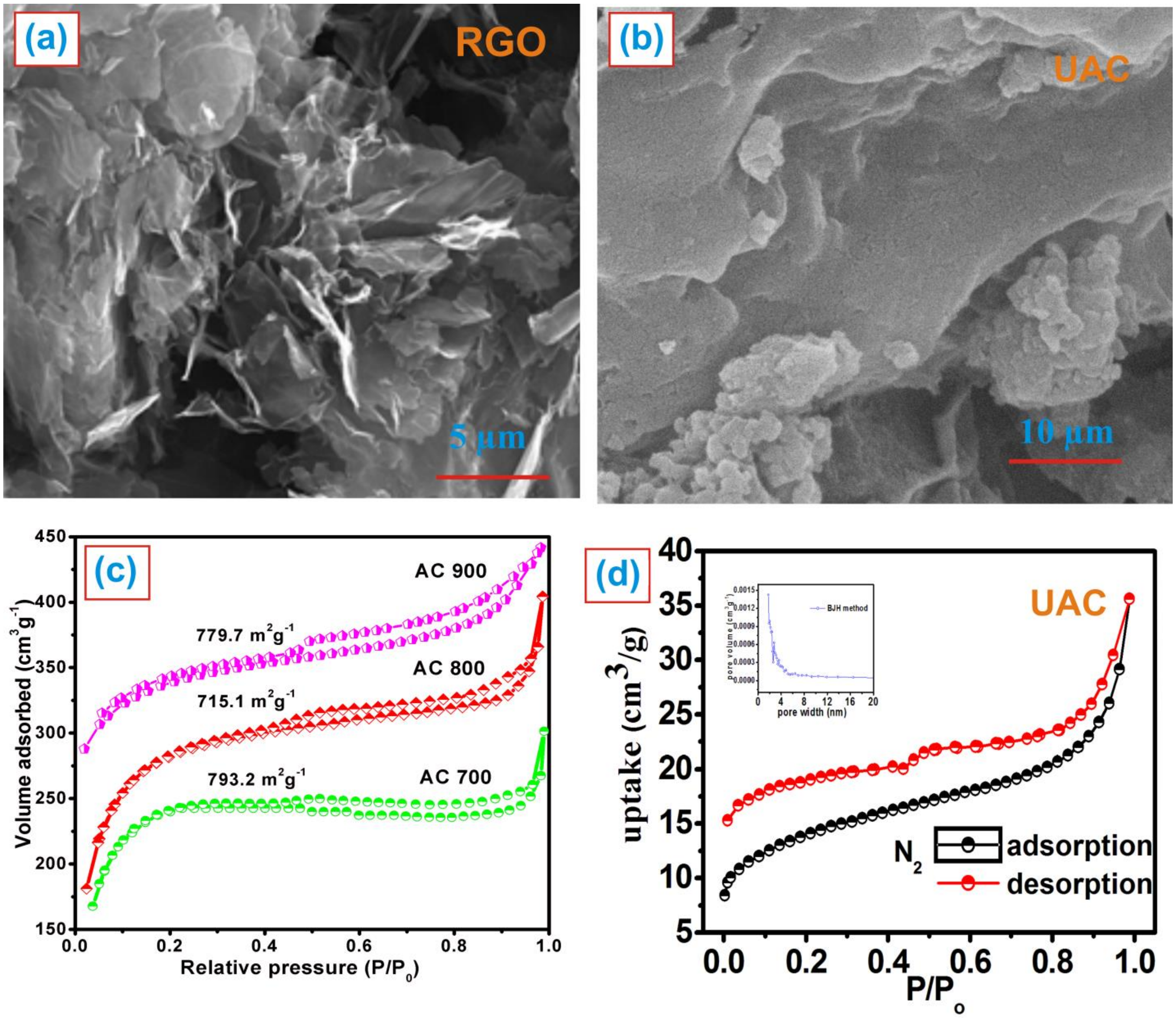

Fig. S1 (a,b) FE-SEM images of RGO and UAC. (c,d) N_2 adsorption−desorption isotherms of ACs and UAC (inset:pore size distribution).

Figure S2

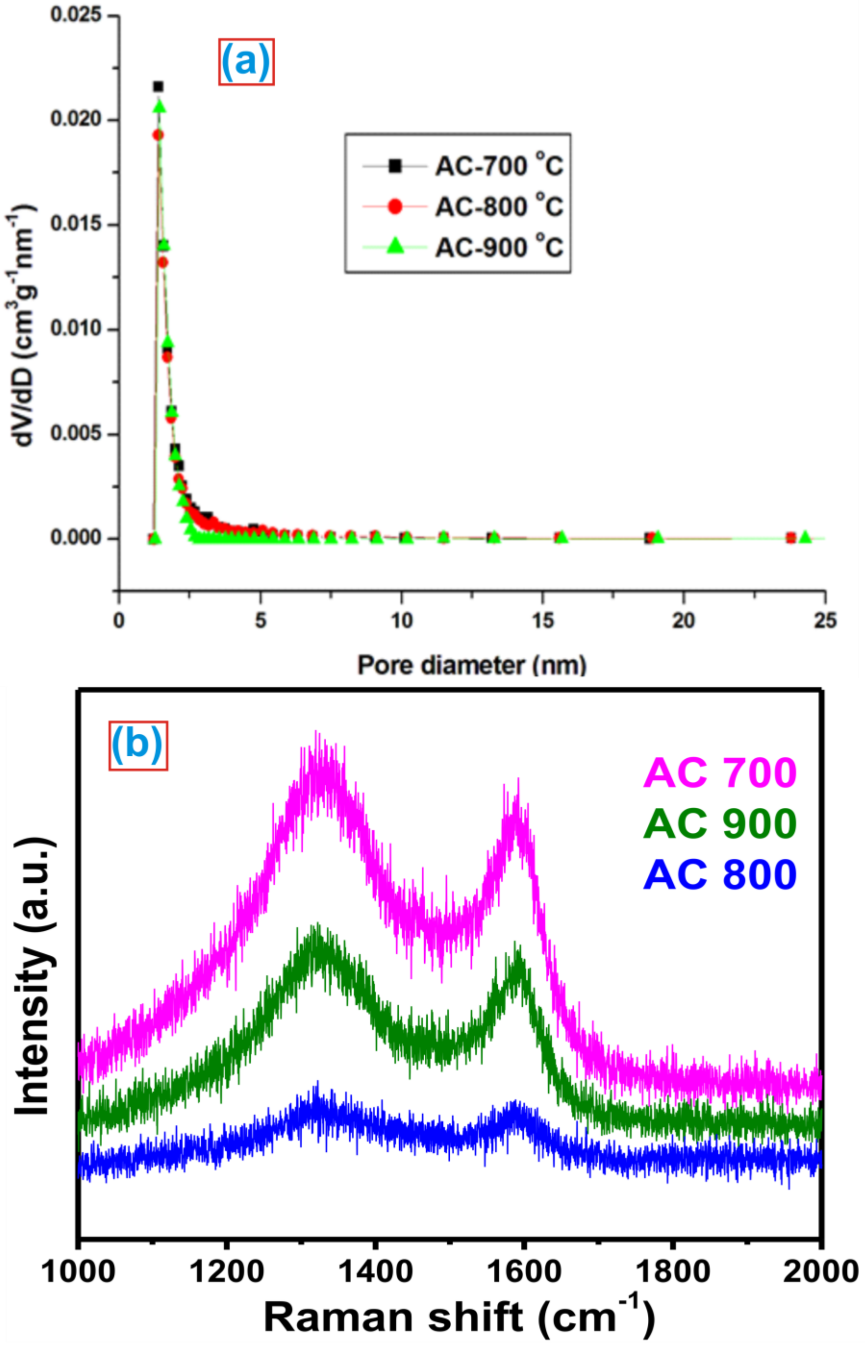

Fig. S2 (a) Pore-size distributions of ACs, (b) Raman spectra of ACs.

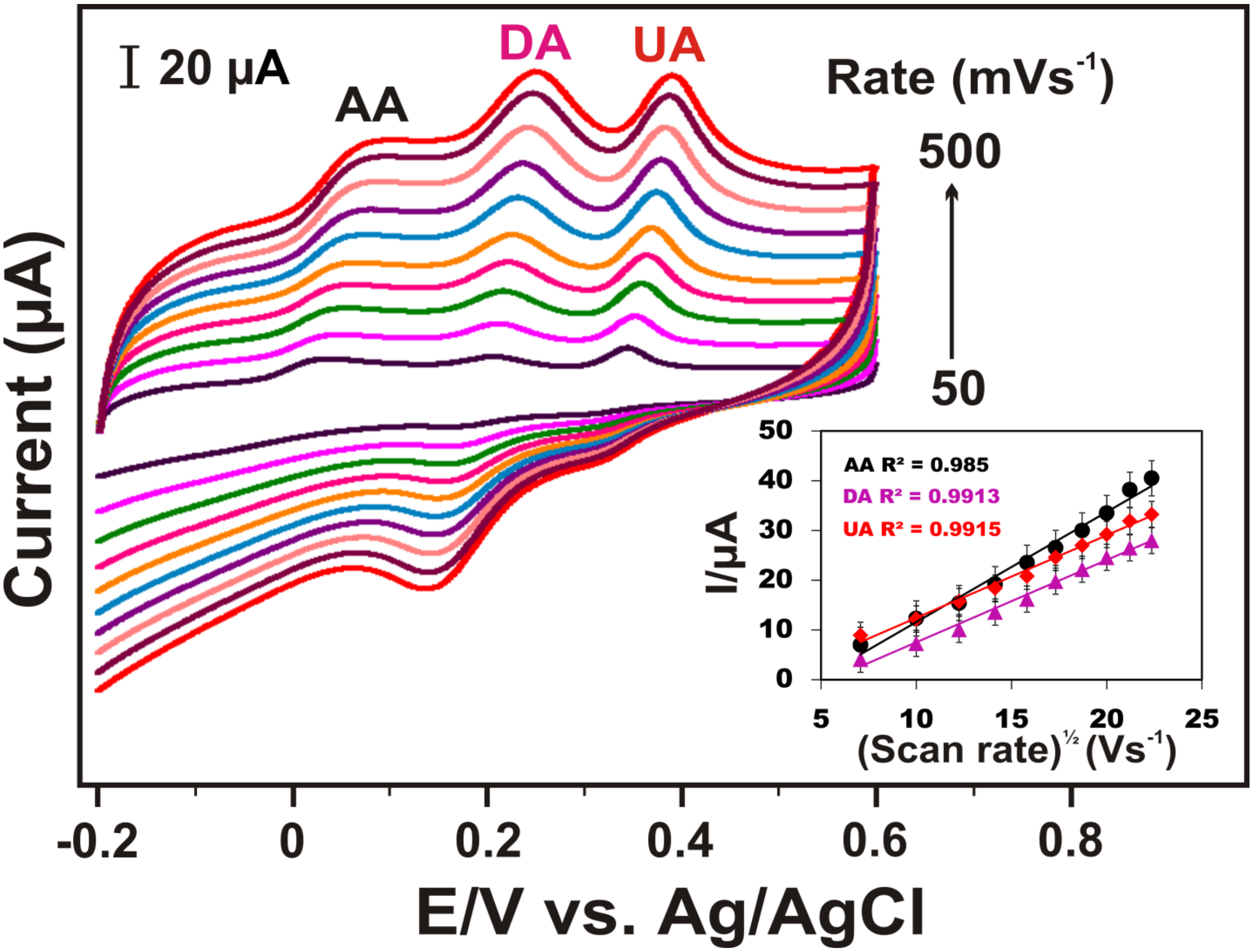

Fig. S3 CV curves of AC700-modified GCE in the presence in 0.1 M PBS (pH 7), which contained a mixture of 30 µM AA + 10 µM DA + 20 µM UA concentrations recorded at different scan rates ranging from 50 to 500 mVs^{-1}. Inset: correlations between peak currents and square root of scan rate.

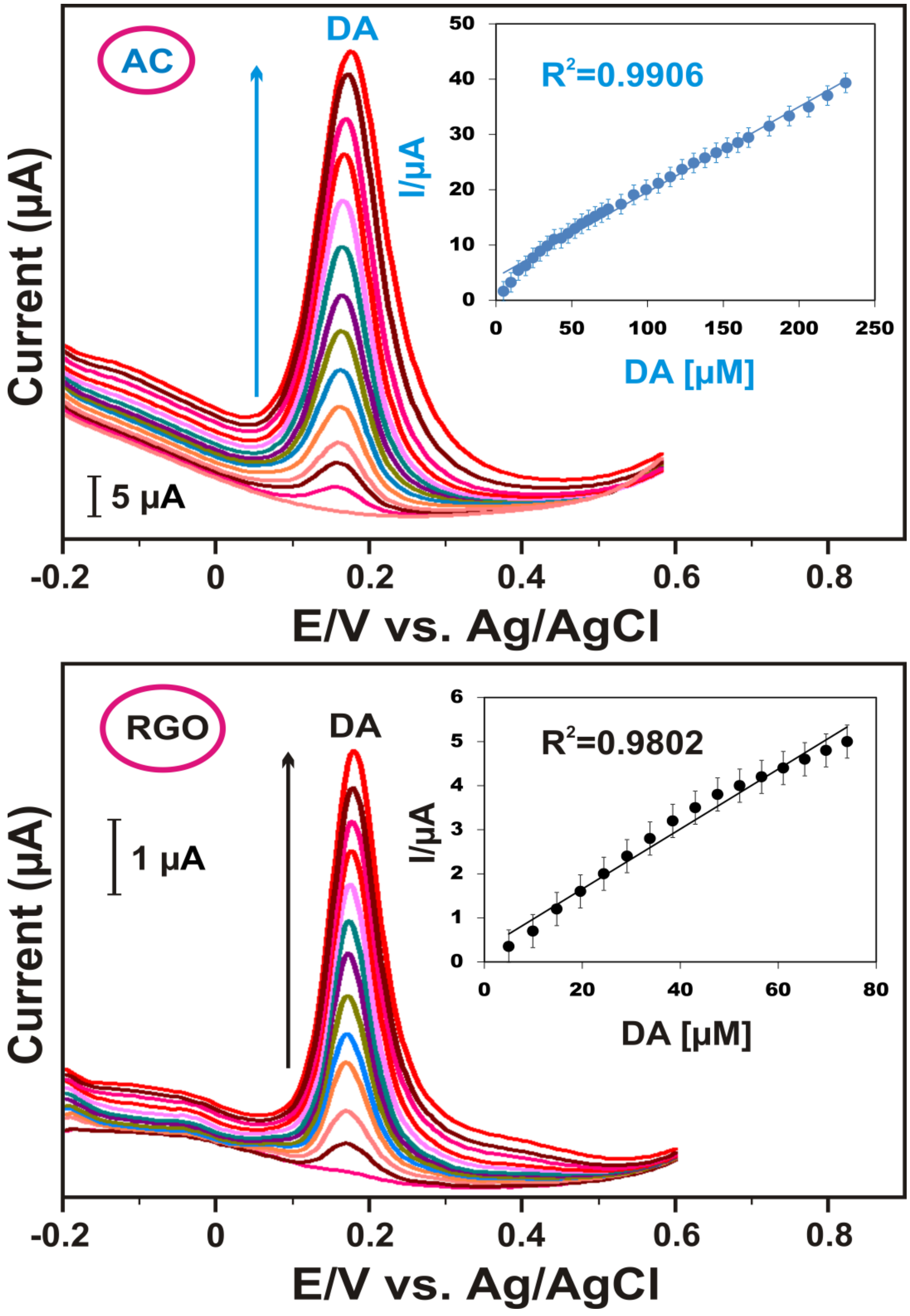
AC
DA
R²=0.9906
I/μA
DA [μM]
5 μA
Current (μA)
E/V vs. Ag/AgCl
RGO
DA
R²=0.9802
I/μA
DA [μM]
1 μA
Current (μA)
E/V vs. Ag/AgCl

Fig. S4 (a) DPV curves of AC700, (b) RGO modified GCE under varied DA concentrations (5 µM-230 µM) and (5 µM-115 µM), respectively in 0.1 M PBS (pH 7). Insets; anodic oxidation peak current *vs* biomolecules concentration.

Table S1: CHNS Elemental Analysis of AC 700 sample.

**AC700 Weight (mg)**	**C atom/%**	**N atom/%**	**S atom/%**	**H atom/%**
**2.206**	83.465	1.074	0.386	0.873
**2.114**	83.389	1.096	0.380	0.849
**Mean**	**83.427**	**1.085**	**0.383**	**0.861**

C – carbon, N – nitrogen, S – sulfur, H – hydrogen.

Table S2: Analytical parameters of the biomolecules at our AC modified electrode and comparison of DA catalytic performance with RGO.

**Method of detection**	**Analytes**	**Limit of detection (μM)**	**Linear range (μM)**	**Correlation coefficient**	**Sensitivity ($\mu A\ \mu M^{-1} cm^{-2}$)**
Selective analysis, AC	AA	4.96	30-95	0.9849	3.5
	DA	0.06	1-65	0.9946	15
	UA	0.75	2-230	0.9903	4.1
Simultaneous analysis, AC	AA	2.3	4-99	0.9834	7.6
	DA	0.03	1-74	0.9928	6.3
	UA	0.51	2-74	0.9966	6.1
Selective AC	DA	0.045	5-230	0.9906	2
Selective RGO	DA	0.5	5-115	0.9802	0.76

Table S3 Real sample analysis for the detection of biomolecules over the AC-modified electrode obtained from DPV measurements

**Real samples**	**Analytes**	**Added (μM)**	**Found (μM)**	**Recovery (%)**
**Human Blood Serum**	AA	0	-	-
		25	24.8	99.2
		50	48.2	96.4
	DA	0	-	-
		25	23.8	95.2
		50	48.2	96.4
	UA	0	1.2	-
		25	25.5	102
		50	52.2	104.4
